# Characteristics of ED crowding in the Lazio Region (Italy) and short-term health outcomes

**DOI:** 10.1007/s11739-018-1881-3

**Published:** 2018-05-25

**Authors:** Francesca Mataloni, Luigi Pinnarelli, Carlo Alberto Perucci, Marina Davoli, Danilo Fusco

**Affiliations:** 1 Department of Epidemiology, Lazio Regional Health Service, Via Cristoforo Colombo, 112, 00147 Rome, Italy; 2 Senior Epidemiologist Consultant, Rome, Italy

**Keywords:** Emergency department, Crowding, Mortality, Hospitalization, Discharge, Length of stay

## Abstract

The effect of emergency department (ED) crowding on patient care has been studied for several years in the scientific literature. We evaluate the association between ED crowding and short-term mortality and hospitalization in the Lazio region (Italy) using two different measures. A cohort of visits in the Lazio region ED during 2012–2014 was enrolled. Only discharged patients were selected. ED crowding was estimated using two measures, length of stay (LOS), and Emergency Department volume (EDV). LOS was defined as the interval of time from entrance to discharge; EDV was defined at the time of each new entrance in ED. The outcomes under study were mortality and hospitalization within 7 days from ED discharge. A multivariate logistic model was performed (Odds Ratios, ORs, 95% CI). The cohort includes 2,344,572 visits. ED crowding is associated with an increased risk of short-term hospitalization using both LOS and EDV as exposures (LOS 1–2 h: OR = 1.71, 95% CI 1.66–1.76, LOS 2–5 h: OR = 1.38, 95% CI 1.34–1.43, LOS > 5 h OR = 1.45 95% CI 1.40–1.50 compared to patients with 1 h of LOS; EDV 75°–95° percentile: OR = 1.02, 95% CI 0.99–1.05 and EDV > 95° percentile: OR = 1.06, 95% CI 1.01–1.11 compared to patients with a EDV < 75° percentile upon arrival). Increased risk of short-term mortality is found with increasing level of LOS. High levels of EDV at the time of patients’ arrival and longer LOS in ED are associated with greater risks of hospitalization for patients discharged 7 days before. LOS in ED is also associated with an increased risk of mortality.

**Electronic supplementary material:**

The online version of this article (10.1007/s11739-018-1881-3) contains supplementary material, which is available to authorized users.

## Introduction

Emergency department (ED) crowding is considered to be one of the key factors that hampers the delivery of high-quality emergency care. ED performance is often evaluated using measures of crowding (e.g., wait times, length of visit, or the proportion of patients seen within their triage target timeframe) [1–3], which represents a very complex problem concerning the balance between demand and supply for emergency services.

The possible effect of ED crowding on patient health has been assessed in several papers [4–16] and systematic reviews [17–20]. Some studies show that most adverse events are preventable, and often related to diagnostic or management errors [21]. Excesses in short-term mortality, hospitalization, transport delays or treatment delays are found [12–16]. Different types of study populations were considered; many studies analyzed all ED patients [11, 13, 16], while others focused on a specific type of patient or condition [7–10, 14]. There is no measure of ED crowding that is universally recognized in the scientific literature, so this phenomenon has been defined using different types of measures. Most of the published studies have considered length of stay in the ED, assuming that the longer the length of stay, the greater the level of ED crowding [6, 8–10, 13]. Others studies have analyzed ED occupancy, ED volume or ambulance diversion as measures of ED crowding [5, 11, 12, 14]. Some studies have estimated crowding using several parameters [15, 16, 21]. ED crowding is a problem that affects health care systems in many countries, but the cause of this phenomenon is still controversial. Many studies have linked ED crowding with the tendency of patients to visit EDs for symptoms or conditions that could be managed by a general practitioner [22, 29], and on the contrary, other researchers have considered this point of view as simplistic, because it does not address the multidimensional and complex causes of the problem [30]. To limit adverse consequences caused by ED crowding and to address the problem, national guidelines have been defined in many European and American countries [23–25]. In some cases, policies and protocols have been adopted to solve this problem, and their impact has been evaluated by the literature [29].

In Italy, there are no such guidelines; thus, hospital administrators approach these challenges using many differing approaches. The Italian National Health Service is based on the principles of universal coverage, and provides healthcare basket benefits homogenously within the national territory. Healthcare is provided to all citizens and residents by a mixed public–private system. The public part is the national health service, Sistema Sanitario Nazionale (SSN), which is financed through the use of general taxation. The SSN is responsible for the Emergency medical services (EMS), which are under Public Health Authorities control in each Italian Region, and the provision of EMS has been undertaken by the local hospital equipped with an ED. Some hospitals often provide a single type of service, such as neonatal or eye-related emergencies, while others provide the full range of EMS. Each patient is evaluated at the check-in desk (Triage) and assigned a color in accordance with his level of need: RED for life-threatening conditions, YELLOW for potentially life-threatening conditions, GREEN for minor injuries or illnesses, or WHITE for non-urgent conditions. Italian hospitals must record all the admissions and ED visits into the national Health Information Systems to receive payment for healthcare services. The Italian Health Information Systems collect all data regarding the healthcare provided by SSN.

The objectives of this study are:

- to describe level of crowding in Lazio Region EDs using two different measures;
- to show characteristics of discharged patients by level of crowding;
- to evaluate the impact of ED crowding on short-term mortality and hospitalization within 7 days after discharge in the Lazio region (Italy).

## Materials and methods

### Data sources

We collected data from the following Italian Health Information Systems: Healthcare Emergency Information System (HEIS), the Hospital Information System (HIS). The HEIS collects information related to all visits to ED of the Region: patient demographics, admission information, visit and discharge dates and hours, ICD-9-CM diagnosis at discharge, reported symptoms on arrival, status at discharge (e.g., dead, hospitalized, or discharged at home) and triage score. Triage score in Italy goes from white (comparable with level 5 in the Emergency Severity Index [31]) to red (comparable to level 1). The HIS is an integrated information system designed to collect clinical and administrative information regarding hospital admissions for each patient discharged from all public and private hospitals of Lazio Region.

Moreover, we collect data from Anagrafe Tributaria, a tax register used to gather fiscal information and to verify the certificate of existence for every patient. Data from the different information systems were merged by a deterministic record linkage procedure based on anonymous identification codes. The HEIS was used to enrol the study cohort (ED patients) and to define their characteristics. Patient comorbidities and hospitalization outcome were extracted from the HIS and mortality information was obtained from the Anagrafe Tributaria.

### Setting and participants

We carried out a retrospective cohort study that included all ED visits from January 1, 2012 to December 31, 2014 in the Lazio, a region of central Italy. In the Lazio region, there are 49 EDs, 22 of which are in Rome; we excluded two high-specialized ED from our study (one dental ED and one ophthalmic ED). Therefore, we considered 47 EDs. We excluded patient visits according to the following criteria:

- not a resident of the Lazio region;
- less than 18 years old;
- diagnosis of delivery (ICD-9-CM codes: V27, 650, 640–676 with 1 or 2 as the fifth number);
- red or missing triage;
- symptoms of shock or coma;
- hospitalized, dead, transferred patients, who have refused hospitalization or have left the ED without being seen;
- hospitalized the day after the visit;
- length of stay over 24 h.

Only the visits of patients who were seen by a physician and discharged were considered in the analysis. Patients who were hospitalized the day after the visit and who have had a length of stay over 24 h were excluded to avoid planned hospitalizations. Multiple visits by the same patient were analyzed, but if a patient had multiple visits to an ED within 7 days, then only the first one was selected.

### Measures of crowding

ED crowding was defined according to two different measures.

The first one was the length of stay (LOS), which is commonly used in the scientific literature [6, 8–10, 13, 16]. Length of stay was defined at the patient level as the interval of time from arrival until discharge. A patient was considered not exposed to ED crowding if he or she had LOS of less than 1 h from arrival until discharge. In particular, LOS was defined using four categories:

- patients discharged within 1 h (reference category).
- patients discharged between 1 and 2 h;
- patients discharged between 2 and 5 h;
- patients discharged after 5 h.

The second measure of crowding was based on ED volume (EDV). EDV was defined at the time of each new visit to the ED. Each patient was considered exposed or not on the basis of the number of patient present in the ED at his arrival:

- number of patients lower than 75th percentile (reference category);
- number of patients between 75th and 95th percentile (crowded);
- number of patients greater than 95th percentile (overcrowded).

ED crowding cut-off of 75° and 95° percentile was defined on the basis of the distribution of patients, present in ED, minute by minute for the period 2012–2014 and specific for each ED and three time bands (8:00–15:59, 16:00–23:59 or 00:00–7:59). EDV cut-points were chosen a priori; level of crowding below the 75° percentile represents the management of a normal situation, then two levels of crowding were defined above the 75° and 95° percentiles to identify one level of crowding and one level of extreme crowding.

### Risk factors and health outcomes

For the third aim, we evaluated the impact of ED crowding in terms of mortality and hospitalization within 7 days from ED discharge. If a patient was hospitalized within 3 days from the discharge, and then died within 7 days from the discharge, we considered both outcomes as interest.

For each patient in the cohort, the risk factors potentially associated with the study outcome were defined. Gender, age, comorbidities, time band, weekend/holiday vs. weekday, previous number of visits to the ED, reported symptoms at the time of arrival and triage score were identified as confounders. Comorbidities were identified by ICD-9-CM codes (details and codes were reported in the Online Resource 1) that were registered at previous hospital admissions during the last 5 years, while previous visits to the ED over the last 5 years were extracted from the HEIS database and classified as 0, 1–3, 4–6, and > 6 visits. When a patient arrives at the ED, he or she is asked to describe their perceived symptoms, and a triage score (white, green, yellow, or red) is assigned; this information was used to characterize the severity of the patient’s condition. The hour of arrival to ED was categorized in three time bands (day 8:00–15:00, evening 16:00–23:00, or night 00:00–7:00), also the day of arrival was categorized in weekend/holiday vs. weekday.

### Statistical analysis

To determine the association between ED crowding and short-term mortality and hospitalization, we used logistic regression models (odds ratios, (ORs), and 95% CIs) with LOS and EDV as exposures. The models were adjusted for gender, age, comorbidities (as dummy variables), time band, weekend/holiday vs. weekday, number of visits to the ED in the past 5 years, the reported symptoms at the time of arrival and triage score.

The SAS (SAS Institute Inc., North Carolina) software program was used for statistical analysis.

## Results

**Fig. 1:**
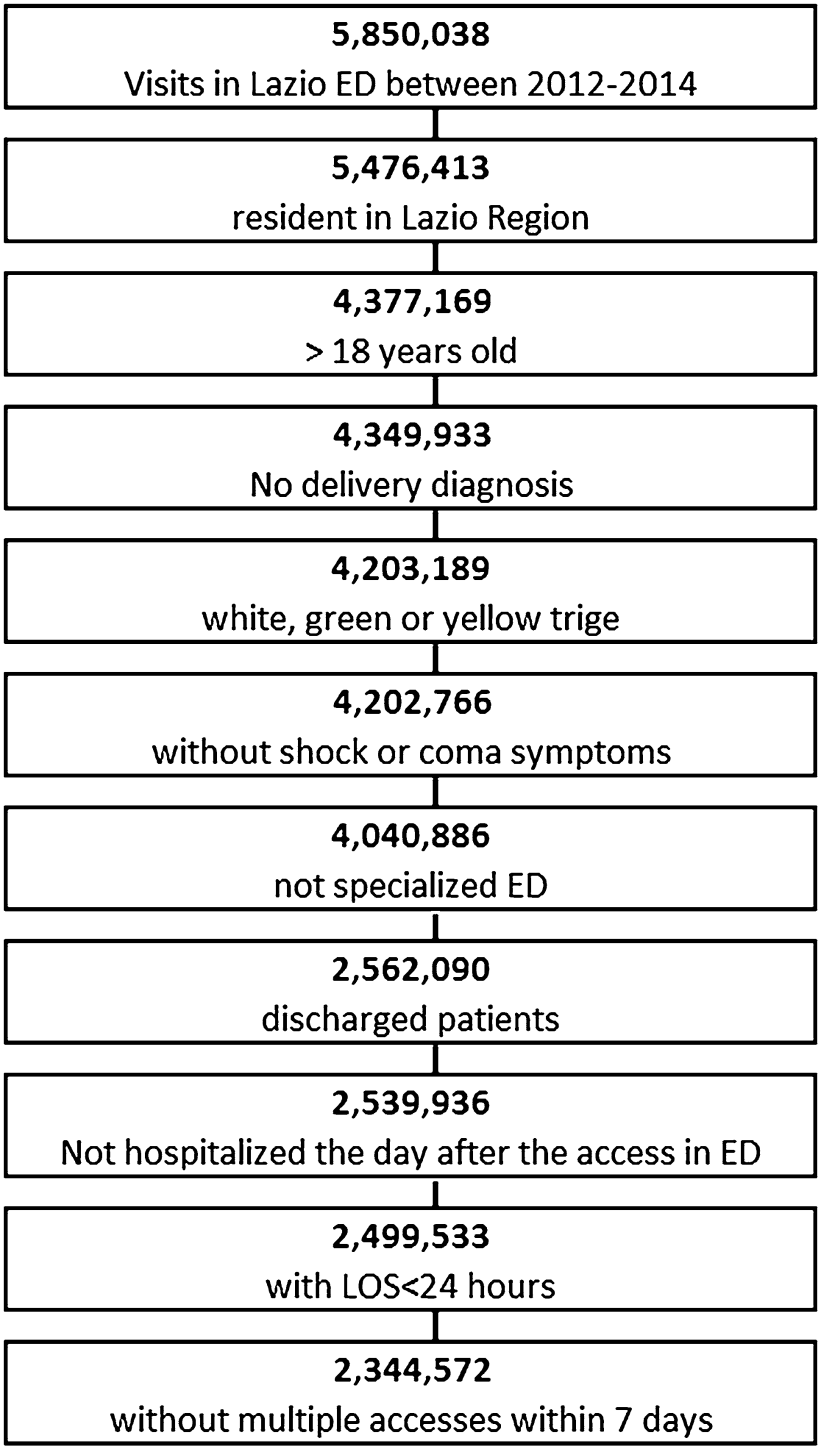
Flow chart of cohort enrolment

In Fig. 1, the exclusion criteria for the study cohort were reported.

There were 5,850,038 visits to Lazio EDs during the study period, of which 1,472,869 involved patients younger than 18 years old or who were not residents of the region. 3% of the visits were excluded because they involved a diagnosis of delivery, a red or missing triage or symptoms of shock or coma. Of the remaining 4,202,766 visits, 161,880 were to the two specialized EDs of the region. More than 25% of the visits were excluded because only visits of patients who had been discharged were considered; 22,154 patients were hospitalized the day after their visit, and 40,403 visits had a length of stay in the ED that was greater than 24 h. Finally, 152,992 visits were excluded because they involved a patient who returned within 7 days. The study cohort consisted of 2,344,572 visits to the ED by patients who were seen and discharged.

Characteristics of ED crowding measures are shown in Table 1.

**Table 1:** Characteristics of ED crowding measures (EDLOS and EDV) by ED volume, ED area and time-band, in the cohort of discharged patients: mean, standard deviation and percentiles

Measure of crowding			Mean	Standard deviation	25°‰	50°‰	75°‰	95°‰
EDLOS (hour)	Total		3.1	3.3	1.1	2.1	3.9	9.2
	ED volume (visits in 1 year)	< 25,000	2.1	2.2	0.8	1.5	2.7	5.6
		> = 25,000	3.2	3.4	1.1	2.2	4.0	9.6
	ED area	Rome	3.3	3.6	1.1	2.2	4.1	10.1
		Rome province	3.1	3.1	1.2	2.3	3.9	8.6
		Others provinces	2.7	2.9	1.0	1.9	3.4	7.9
	Time-band of access	8:00–15:00	3.1	3.1	1.2	2.2	3.9	8.4
		16:00–23:00	3.0	3.6	1.0	1.9	3.6	11.4
		00:00–7:00	3.2	3.3	0.9	2.2	4.4	10.1
EDV (patients)	Total		21.9	18.6	8	16	32	59
	ED volume (visits in 1 year)	< 25,000	6.4	4.2	3	6	9	14
		> = 25,000	24.0	18.7	10	19	34	61
	ED area	Rome	29.5	19.9	14	26	42	67
		Rome province	10.4	7.4	5	9	15	24
		Others provinces	15.6	14.4	5	11	21	47
	Time-band of access	8:00–15:00	24.1	19.3	10	18	35	63
		16:00–23:00	21.2	17.6	8	16	31	57
		00:00–7:00	11.7	12.9	2	7	18	39

On average the study cohort have a length of stay in ED greater than 3 h with a 95° percentile greater than 9 h. EDs with low level of activity (ED Volume) have lower LOS compared to greater ones (2.1 vs. 3.2 h) as happens for an ED sited in Rome compared to others. Mean level LOS did not vary by time-band, patients on average wait 3 h from the access to the discharge regardless the time of access during the day. Characteristics of EDV were expressed in number of patients present in ED at each new arrival. On average there are 22 patients in the ED when a new patient arrives. EDV differs a lot by ED volume of activity (ED with low volume of visits: 6.4; ED with high level of visits: 24). This measure of crowding seems also to be dependent as to where the ED is sited, in fact it is higher in an ED sited in Rome compared to the rest of the region (29.5 patients in EDs of Rome vs. 10.4 in the province of Rome and 15.6 in the rest of the Region). EDV is dependent also to the time at which patients arrive in the ED (time-band); patients who arrive in the morning (8:00–15:00) find on average 24 patients who are waiting for the visit or for the discharge; on the contrary, patients who arrive during the night (00:00–7:00) find, on average, 11.7 patients in the ED.

The main characteristics of the cohort and of the patients who were the most exposed to each measure (LOS and EDV) are described in Table 2. The cohort distributions for both exposures were reported in the Online Resources 2 and 3.

**Table 2:** Characteristics of the study cohort for both exposures (LOS and EDV)

Total	Total		LOS > 5 h		EDV > 95th‰	
	*n*	%	*n*	%	*n*	%
	2,344,572	100	362,717	100	92,524	100
Gender						
Female	1,221,065	52.1	187,958	51.8	49,320	53.3
Male	1,123,507	47.9	174,759	48.2	43,204	46.7
Age class						
18–30	483,459	20.6	49,791	13.7	19,213	20.8
31–50	911,648	38.9	116,160	32.0	35,389	38.2
51–70	574,012	24.5	101,924	28.1	22,909	24.8
> 70	375,453	16.0	94,842	26.1	15,013	16.2
Triage						
Yellow	446,107	19.0	140,146	38.6	22,065	23.8
Green	1,778,033	75.8	216,770	59.8	66,968	72.4
White	120,432	5.1	5,801	1.6	3,491	3.8
Weekend/holiday						
Yes	705,576	30.1	97,346	26.8	13,813	14.9
No	1,638,996	69.9	265,371	73.2	78,711	85.1
Time band of access						
8:00–15:00	1,317,126	56.2	201,563	55.6	52,231	56.5
16:00–23:00	796,187	34.0	112,198	30.9	31,075	33.6
00:00–7:00	231,259	9.9	48,956	13.5	9,218	10.0
Comorbidities						
Malignant cancers	92,070	3.9	21,620	6.0	3,638	3.9
Diabetes	78,409	3.3	21,552	5.9	3,163	3.4
Obesity	26,703	1.1	6,020	1.7	1113	1.2
Hematological diseases	71,081	3.0	18,977	5.2	2928	3.2
Hypertension	257,559	11.0	66,676	18.4	10,127	10.9
Ischemic heart disease	83,735	3.6	25,956	7.2	3348	3.6
Heart failure	41,635	1.8	13,512	3.7	1639	1.8
Arrhythmias	126,129	5.4	36,379	10.0	5073	5.5
Cerebrovascular diseases	74,815	3.2	21,640	6.0	3089	3.3
Vascular diseases	43,478	1.9	11,820	3.3	1727	1.9
COPD	64,442	2.7	18,121	5.0	2565	2.8
Chronic diseases (liver, pancreas, intestine)	37,882	1.6	9013	2.5	1497	1.6
Anemia and coagulopathy	52,738	2.2	14,429	4.0	2205	2.4
Others	168,070	7.2	46,091	12.7	6700	7.2
Symptoms						
Nervous system	30,040	1.3	8370	2.3	1233	1.3
Abdominal pain	151,043	6.4	38,274	10.6	5649	6.1
Chest pain	63,476	2.7	31,840	8.8	2796	3.0
Dyspnea	25,080	1.1	7441	2.1	968	1.0
Nontraumatic hemorrhage	28,964	1.2	4142	1.1	1158	1.3
Trauma or burns	856,762	36.5	74,174	20.4	35,484	38.4
Poisoning, allergic reaction	18,942	0.8	2258	0.6	675	0.7
Fever	23,862	1.0	5615	1.5	945	1.0
Heart rhythm, hypertension	37,675	1.6	11,078	3.1	1541	1.7
Eye, ear, nose, skin	119,725	5.1	4454	1.2	4749	5.1
Genitourinary and obstetric	144,782	6.2	8896	2.5	6306	6.8
Others	843,327	36.0	166,072	45.8	30,980	33.5
Administrative issue	894	0.0	103	0.0	40	0.0
Number of previous visits						
0	594,378	25.4	92,495	25.5	23,638	25.5
1–3	1049,707	44.8	159,078	43.9	41,424	44.8
4–6	387,426	16.5	60,069	16.6	15,239	16.5
> 6	313,061	13.4	51,075	14.1	12,223	13.2

52% of the cohort was female, and the gender distribution did not change for the two exposures. The age distribution of the patients, who were considered to have been exposed to the greatest level of EDV, was similar to that of the total cohort, while the patients exposed to the greatest level of LOS were older (26% > 70 years old vs. 16% of the total cohort). The 75.8% of the cohort was assigned a green triage code. According to triage, patients with longer LOS had more serious conditions compared to the total cohort (yellow triages 38.6 vs. 19%). ED crowding seemed to be less likely during the weekend or on holidays than during weekdays when measured by EDV. The most common comorbidity of the cohort was hypertension (11%), and in general, patients who had waited more than 5 h in the ED seemed to have a worse medical case. The majority of the patients had declared symptoms of trauma or burns (36.5%) or unspecified symptoms (“others”) (36%). A total of 44.8% of the cohort had between 1 and 3 previous visits in the past 5 years.

Table 3 shows the results of the associations between ED crowding (measured with LOS and EDV) and short-term hospitalization and mortality.

**Table 3:** Associations between level of crowding (LOS and EDV) and short term mortality and hospitalization: number of visits (*n*), number of outcome (outcome), Crude Odds Ratio (Crude OR), Adjusted Odds Ratio (adj OR) and 95% confidence intervals (95% CI)

LOS in ED	*N*	Outcome	Crude OR	95% CI	Adj OR	95% CI
Hospitalization						
< 1 h	577,227	11,464	1		1	
1–2 h	832,983	14,668	1.68	1.63–1.73	1.71	1.66–1.76
2–5 h	362,717	7798	1.49	1.45–1.53	1.38	1.34–1.43
> 5 h	571,645	6800	1.83	1.77–1.89	1.45	1.40–1.50
Mortality						
< 1 h	571,645	185	1		1	
1–2 h	577,227	307	1.64	1.37–1.97	1.15	0.96–1.38
2–5 h	832,983	900	3.34	2.85–3.91	1.41	1.20–1.66
> 5 h	362,717	880	7.51	6.41–8.80	1.78	1.51–2.11
EDV	*n*	Outcome	Crude OR	95% CI	Adj OR**	95% CI
Hospitalization						
< 75th percentile	1,842,420	31,601	1		1	
75th–95th percentile	409,628	7387	1.05	1.03–1.08	1.02	0.99–1.05
>95th percentile	92,524	1742	1.10	1.05–1.15	1.06	1.01–1.11
Mortality						
< 75th percentile	1,842,420	1759	1		1	
75th–95th percentile	409,628	412	1.05	0.95–1.17	0.96	0.86–1.07
> 95th percentile	92,524	101	1.14	0.94–1.40	1.03	0.84–1.26

*Adjusted for gender, age, comorbidities, time band, weekend/holiday vs. weekday, number of visits in ED, reported symptoms and triage

The risk of mortality and hospitalization within 7 days from ED discharge was higher for patients who had waited more than 1 h in the ED than for patients in the reference category (for hospitalization: LOS 1–2 h: OR = 1.71, 95% CI 1.66–1.76; LOS 2–5 h: OR = 1.38, 95% CI 1.34–1.43; LOS > 5 h: OR = 1.45 95% CI 1.40–1.50; for mortality: LOS 1–2 h: OR = 1.15, 95% CI 0.96–1.38; LOS 2–5 h: OR = 1.41, 95% CI 1.20–1.66; LOS > 5 h: OR = 1.78 95% CI 1.51–2.11). In the hospitalization analysis, the highest risk was for patients who had a LOS greater than 1 h but less than 2 h. Regardless the statistical significance, patients who arrived at the ED during a crowded condition (when the number of patients in ED was greater than the 75th percentile) had a greater risk of hospitalization within 7 days compared with patients in the reference category (EDV 75th–95th percentile: OR = 1.02, 95% CI 0.99–1.05; EDV > 95th percentile: OR = 1.06, 95% CI 1.01–1.11). There is no evidence of an association between EDV and short-term mortality.

## Discussion

Level of crowding in ED of the Lazio Region was estimated using two measures (EDLOS and EDV) that have different characteristics and units of measure. EDLOS is expressed in hours and EDV in number of patients. We found a positive association between ED crowding, measured using both LOS and EDV, and short-term (7 days) hospitalization. Excesses of mortality risks were found for longer lengths of stay in ED.

Previous studies have investigated the consequences of ED crowding on patient health and treatment, with conflicting results. However, most of the studies reported that worse health outcomes are associated with longer waiting times due to delays in time sensitive treatments for serious conditions [8] or missed diagnoses with the possibility of unnecessary complications [33]. In a study published in 2015, nine measures of crowding were evaluated to investigate whether crowding is associated with higher rates of post-discharge hospitalizations and death [16]. Only the “evaluation time” (time from the first contact with a physician in the ED to the discharge) and the “Total LOS” (time between registration in the ED and the discharge) was associated with an increased risk of hospitalization or death after the discharge. A previous Canadian study analyzed the association of wait times in EDs with short-term mortality and hospital admission in a cohort of discharged patients [13]. They used the mean emergency department length of stay by shift as the exposure and found that the risk of death and hospitalization within 7 days increased incrementally with each additional hour of mean waiting time per shift. Additionally, an Australian study published in 2006 found an effect of ED crowding on patient mortality during different time windows [11]. An increased risk of in-hospital mortality within 10 days of an ED visit was found in one hospital in Australia during a crowded shift compared with a non-crowded one [4]. Hsia et al. hypothesized an association between ED crowding and bounce-back admission in California [12]. Crowded days were identified using ambulance diversion, but an association between ambulance diversion and bounce-back admissions was not found. Several studies analyzed outcomes different from mortality or hospitalization. A 2007 study found that with increasing ED volume, patients affected by pneumonia were less likely to receive timely antibiotic therapy. Delays in analgesic administration to older patients with hip fracture associated with ED crowding were identified in 2006 by Hwang et al. [7]. In contrast, a successive study found that ED crowding did not have any association with a patient’s treatment time [27]. In Ontario patients with mental illness could wait longer than others during not crowded periods but also waited for significantly less time when the level of crowding increased [9].

The scientific literature on ED crowding is abundant and varied, taking several outcomes into account, and different ways to measure emergency department crowding have been considered.

In our study, we used two methods to estimate ED crowding: LOS and EDV. LOS in the ED is one of the most used measures of crowding [6, 8–10, 13, 16, 32]; it was defined at patient level as the difference between time of discharge and arrival to the ED. EDV has been used in other studies [14, 26] where it was generally defined by week or by an 8-h shift; in our study, like a recent one [16], EDV was defined by counting the number of patients present in the ED at the time of each new visit. In this way, each patient arrival in an ED was assigned a specific level of crowding that represented the number of patients who were in that specific ED at arrival. The increased risk of an adverse event after an ED discharge was higher when crowding was measured using the LOS in the ED instead of EDV. Nevertheless, it is difficult to interpret the results generated with LOS as the exposure because the trend of the ORs is not always linear, unlike in other similar studies [9, 13]; this is probably due to the spurious nature of LOS. As was already indicated by Gabayan [16] and as indicated by our results, LOS is not only a measure of ED crowding but also strongly depends on the severity of a patient’s condition. In our cohort, in fact, patients with longer lengths of stay in ED also show more complex and severe health conditions. A patient could wait in an ED for less than an hour for one of two reasons: either he had a high severity condition and needed to be treated quickly or he had a low severity condition and the emergency department was not crowded. Furthermore, a patient could wait more than 5 h if he had a low severity condition and the ED was crowded or if his condition was more severe and he needed more time to be treated. For this reason, we tested EDV as a measure for representing the effective level of crowding by counting the number of patients present in an ED at each patient arrival. The greatest advantages of this measure are that it is ED specific (percentiles of high volume ED are different from low volume ones) and is independent from patient severity. However, the use of EDV could have some limitations. As EDV was defined specifically for each ED and is based on observed data during 2012–2014, the periods of crowding were identified in each ED by definition (> 75° percentile of the specific ED), but in some cases this measure may not truly represent ED crowding that would affect health outcomes. Outcomes under study were mortality and hospitalization within 7 days from ED discharge. Another study considered a time frame of 10 days from an ED visit to the outcome [4]; however, a length of 7 days was considered to be an appropriate period of time to ensure that the event was due to the index ED visit [16].

This is the biggest study to evaluate emergency department crowding in Italy, the results came from a large dataset (more than 2 million ED visits) that included all patients discharged after an ED visit over 3 years (2012–2014) in the Lazio region (Central Italy). The number of ED included in this analysis is also higher than others studies [4, 5, 16, 21] and amounted to 47. A previous Italian study evaluated how overcrowding may affect urgent patients’ waiting times and the extra costs due to inappropriate use of EDs, but is based on only 1 year and patients studied amount to 54,254 [32].

The models were adjusted for several confounders used to characterize the patients (gender and age), their health conditions (previous comorbidities, reported symptoms at the time of arrival and triage score), their tendencies to return to an ED (number of previous visits to an ED) and their visits to an ED (time band and weekend/holiday vs. weekday). Nevertheless, all of this information was derived from administrative data. For this reason, it is not possible to evaluate the quality and completeness of the data, and there could be a potential misclassification of triage, as 75.8% of the cohort was reported as a green code. In addition, the information related to the declared symptoms is not informative; 36% of the cohort reported a symptom classified as “other”. Thus, the comorbidities obtained from a previous hospital admission were used to characterize the patients. Furthermore, we cannot exclude the possibility of residual confounding that may distort the results.

Our future research will be focused on the effect of crowding on the treatment of patients with specific diagnosis and in the evaluation of the different impact of crowding in patient treatment within hospitals.

In conclusion, we find an association between ED crowding and hospitalization and mortality within 7 days of an ED discharge; this association is found only when ED crowding is measured with LOS. The length of stay in an ED, despite its widespread use, is a spurious measurement of crowding, while the number of patients in the ED at any given time represents a better measurement of crowding and is not affected by the severity of a patient’s condition. LOS masks the effect of crowding on health outcomes; therefore, more granular measurements, such as EDV, are preferred because they preserve more information regarding crowding effects on health outcome [28].

## Electronic supplementary material

Below is the link to the electronic supplementary material.

Supplementary material 1 (XLS 28 kb)

Supplementary material 2 (XLS 41 kb)

Supplementary material 3 (XLS 47 kb)
